# Case Report: Local injection of an IL-17 inhibitor successfully treats Acrodermatitis continua of Hallopeau and avoids immune shift

**DOI:** 10.3389/fimmu.2026.1869496

**Published:** 2026-06-09

**Authors:** Yali Li, Shiying Li, Yuning Zhang, Yuhan Chen, Tao Guo, Chen Li

**Affiliations:** 1 Department of Dermatology, Tianjin Academy of Traditional Chinese Medicine Affiliated Hospital, Tianjin, China; 2 Department of Internal Medicine, Tianjin Gong An Hospital, Tianjin, China; 3 Graduate School, Tianjin University of Traditional Chinese Medicine, Tianjin, China; 4 Graduate School, Beijing University of Chinese Medicine, Beijing, China

**Keywords:** Acrodermatitis continua of Hallopeau, case report, IL-17, immune drift, local injection, psoriasis, xeligekimab

## Abstract

Acrodermatitis continua of Hallopeau (ACH) is a refractory, chronic, sterile pustular psoriasis predominantly involving the acral regions. Although biologic agents targeting the interleukin-17 (IL-17) pathway have demonstrated clinical promise, systemic administration may induce eczematous eruptions, known as “immune drift,” due to Th17/Th2 immune imbalance. This report presents a novel and successful case, being the first to describe a patient with refractory ACH who was successfully treated with local injections of an IL-17 inhibitor, thereby avoiding immune drift. A 13-year-old female patient with ACH showed poor responses to systemic acitretin, upadacitinib, and topical calcipotriol/betamethasone. After switching to systemic Xeligekimab, her hand and foot pustules improved rapidly, but eczematous eruptions appeared. Subsequently, the treatment was switched to diluted Xeligekimab administered via divided local injections around the lesions on the hands and feet. By week 4, periungual lesions markedly improved; by week 16, erythema and pustules had largely resolved, and scaling significantly improved. The Palmoplantar Pustulosis Area and Severity Index (PPPASI), Dermatology Life Quality Index (DLQI), and Nail Psoriasis Severity Index (NAPSI) all decreased substantially, and no immune drift recurred. This case suggests that local injection of an IL-17 inhibitor may effectively control skin lesions while potentially avoiding systemic immune imbalance, offering a preliminary novel treatment approach for refractory ACH that requires further validation.

## Introduction

Acrodermatitis continua of Hallopeau (ACH) is a chronic, relapsing, sterile pustular skin disorder predominantly affecting the distal portions of fingers and toes. Clinically, it is characterized by periungual and subungual sterile pustules, paronychia, nail dystrophy, acro-osteolysis, acral pain and atrophy, making its treatment particularly challenging (1). Conventional treatments such as cyclosporine and acitretin have low overall response rates (2). Recent studies have shown that JAK inhibitors (such as upadacitinib) and interleukin-17 (IL-17) inhibitors exhibit certain efficacy in ACH patients (3–5), but issues such as variable responses and therapeutic plateau phases exist. Systemic administration of IL-17 inhibitors may also disrupt immune homeostasis, shifting the Th17/Th2 balance toward a Th2-dominant state, leading to an “immune drift” phenomenon. Here we report a patient with refractory ACH who had poor responses to systemic acitretin, upadacitinib, and topical calcipotriol/betamethasone, and who achieved remarkable clinical efficacy and successfully avoided immune drift after receiving divided local injections of Xeligekimab.

## Case presentation

A 13-year-old Asian female of Han ethnicity was diagnosed with ACH two years prior, based on recurrent erythema, pustules, erosions, and scaling on the hands and feet, accompanied by nail thickening, whitening, and opacification. Oral acitretin (10 mg once daily) and topical calcipotriol/betamethasone ointment for three months were ineffective. The treatment was then switched to oral upadacitinib extended-release tablets (15 mg/day) for six months, which slightly improved hand and foot pustules, but the condition subsequently relapsed and progressed, with expansion of pustules on the palms and soles, further nail thickening and opacity, and partial nail destruction, fragmentation, and oozing of blood. No joint swelling, morning stiffness, or digital bone deformity was observed, and clinical evaluation showed no evidence of psoriatic arthritis or other inflammatory joint diseases. Upon presentation to our hospital, her Palmoplantar Pustulosis Area and Severity Index (PPPASI) was 14.4, the Dermatology Life Quality Index (DLQI) was 24, and the Nail Psoriasis Severity Index (NAPSI) was 160. The patient experienced significant pain, severely affecting her quality of life. She was treated with a standard dose (100 mg/mL) of Xeligekimab subcutaneously. The next day, the pustules on her hands and feet slightly improved, but diffuse eczematous eruptions appeared all over the body, predominantly on the face. Topical calcineurin inhibitors resolved the eruptions. To avoid recurrence of immune drift, we adopted a low-concentration, multi-site local injection strategy. Specifically, 0.2 mL of Xeligekimab (100 mg/mL) was diluted with 0.8 mL of normal saline to achieve a 5-fold dilution (20 mg/mL). Then, 0.1 mL (2 mg) was injected at six sites on the hands and feet, avoiding vascular plexuses and severely affected areas, resulting in a total dose of 12 mg per treatment session. Injections were administered every two weeks (6) (Supplementary Figure).

## Results

**Figure 1:**
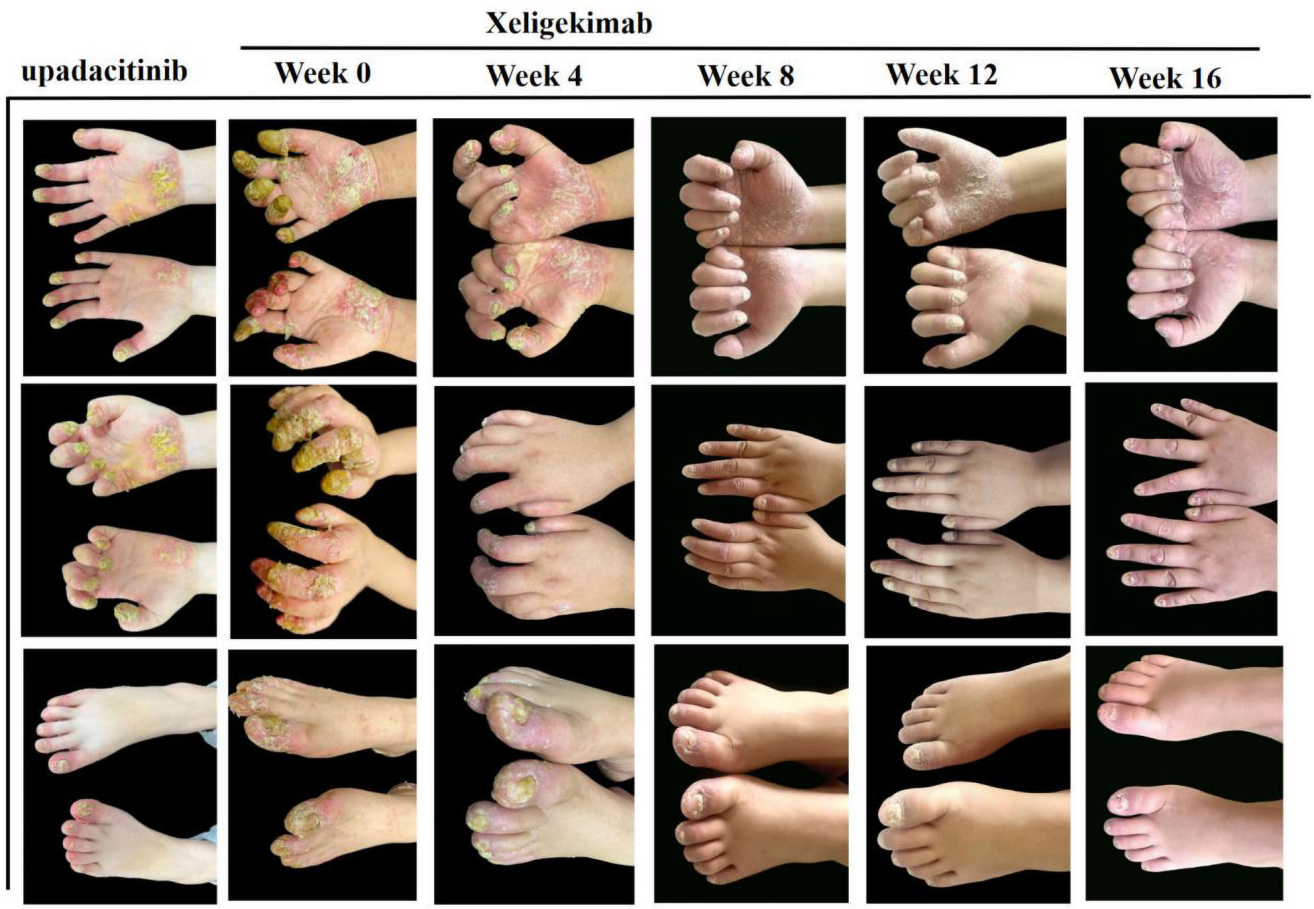
The clinical manifestations of the patient were assessed before upadacitinib treatment and at 0, 4, 8, 12, and 16 weeks after initiation of Xeligekimab therapy.

**Figure 2:**
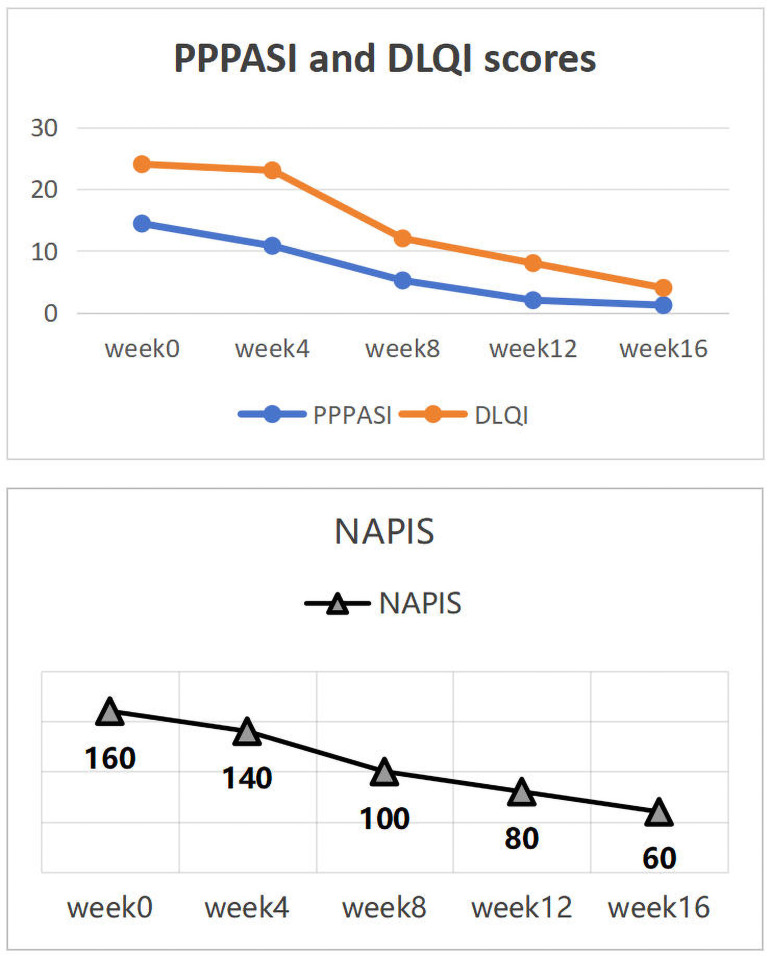
Changes in DLQI, PPPASI and NAPIS at Weeks 0, 4, 8, 12 and 16 of Xeligekimab treatment.

**Figure 3:**
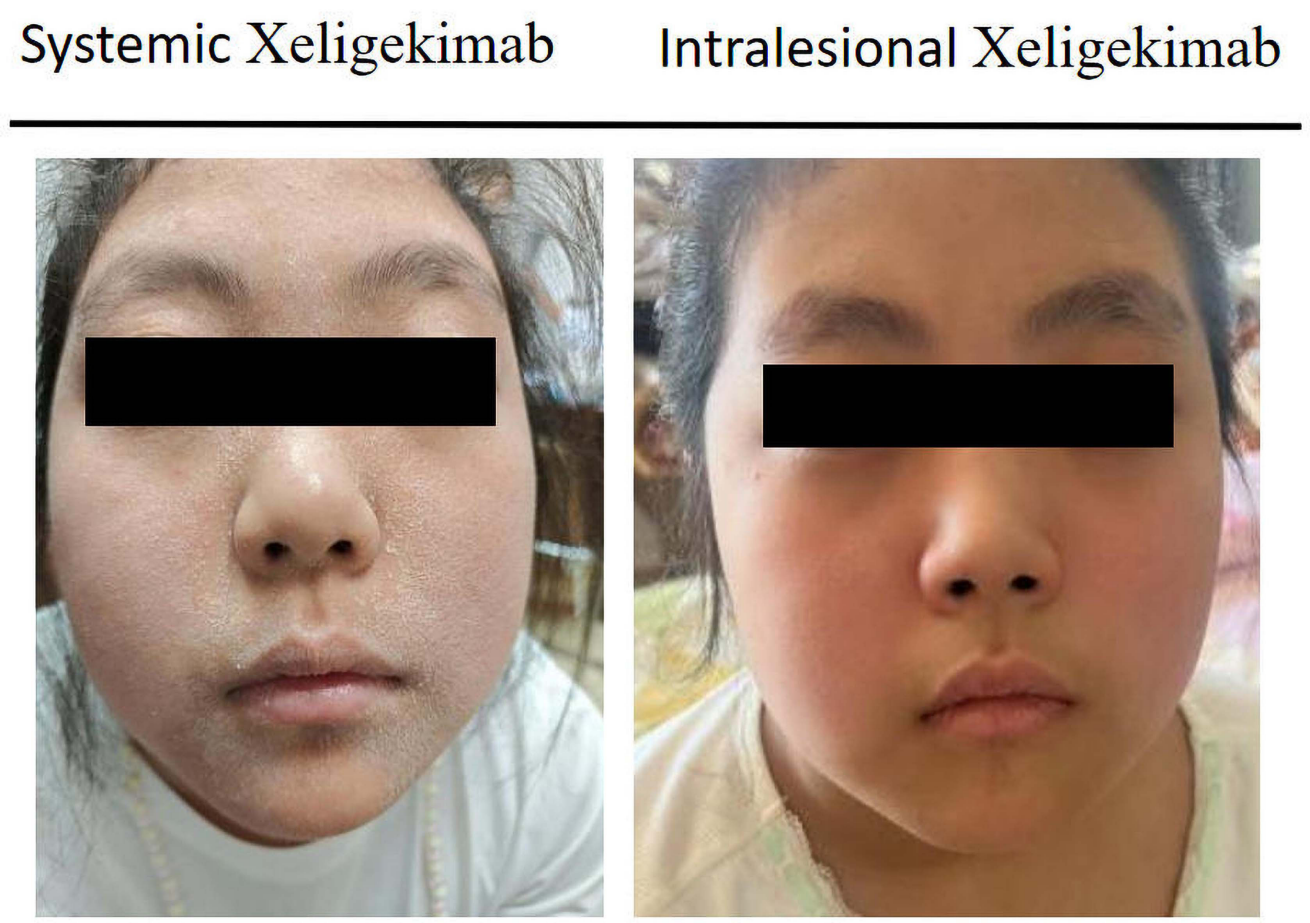
Clinical response to different routes of Xeligekimab administration.

After 16 weeks of treatment, pustules on the patient’s hands and feet resolved completely, with no nail bed bleeding or periungual purulent discharge, leaving some nail shedding and dystrophy of the fingernails and toenails (**Figure 1**). From week 4 to week 16, PPPASI, DLQI, and NAPSI continuously improved: PPPASI decreased from baseline 14.4 to 1.2, DLQI from 24 to 4, and NAPSI from 160 to 60 (**Figure 2**). The patient experienced no recurrence of eczema or any systemic adverse reactions (**Figure 3**).

## Discussion

Acrodermatitis continua of Hallopeau, palmoplantar pustulosis (PPP), and generalized pustular psoriasis (GPP) all belong to the spectrum of pustular psoriasis. Unlike the diffuse systemic aseptic pustules, fever, and general toxic symptoms of GPP, ACH is a localized pustular psoriasis with nail involvement, mainly affecting the distal fingers and toes, and follows a chronic, relapsing course. The pathogenesis of ACH is not fully understood, but current research suggests that genetic susceptibility and aberrant activation of the innate immune system play important roles. Firstly, in terms of genetic susceptibility, loss-of-function mutations in the interleukin-36 receptor antagonist (IL36RN) gene are common susceptibility factors for both GPP and ACH. The IL36RN gene encodes the IL-36 receptor antagonist (IL-36Ra); loss-of-function mutations can lead to overactivation of the IL-36 signaling pathway. IL-36, a member of the IL-1 family primarily produced by epithelial cells such as keratinocytes, drives ACH pathogenesis when its signaling is dysregulated. Secondly, overactivation of IL-36 not only directly promotes neutrophil chemotaxis and aggregation in the epidermis, forming characteristic Kogoj spongiform pustules, but also induces expression of downstream pro-inflammatory cytokines (e.g., IL-1, IL-17, IL-23, and TNF-α), thereby driving abnormal keratinocyte proliferation and differentiation (7–9). Among these, IL-17 further induces keratinocytes to secrete antimicrobial peptides and chemokines, recruiting more neutrophils and establishing a positive feedback loop of neutrophil–keratinocyte interaction that amplifies local inflammation (10, 11). Given the central role of the IL-36 pathway in the pathogenesis of pustular psoriasis, IL-36 receptor inhibitors (such as spesolimab) may provide a reasonable alternative or salvage treatment option for refractory ACH patients (12). At the signal transduction level, aberrant activation of the JAK/STAT (Janus Kinase/Signal Transducer and Activator of Transcription) pathway has also been confirmed as an important mechanism in ACH, and clinical improvement with JAK inhibitors supports this.

Upadacitinib, a JAK1 inhibitor, exerts anti-inflammatory and tissue-repair effects by inhibiting STAT-mediated inflammatory pathways. Studies have shown that JAK inhibitors have good efficacy in localized pustular psoriasis (13). Han et al. first reported a patient with refractory ACH who, after switching to a JAK inhibitor, showed significant improvement in skin and joint symptoms within 20 weeks, and imaging revealed no progression of bone erosion, suggesting that JAK inhibitors may delay ACH-associated bone pathology (14). In the present case, the patient’s response to upadacitinib was consistent with some literature reports, with gradual improvement of skin lesions. However, all current evidence for JAK inhibitors in ACH comes from case reports and small retrospective analyses, with low levels of evidence and limited generalizability. Although doses of 30 mg/day have been reported in adult refractory cases, the optimal dose of upadacitinib in adolescent ACH patients has not been established. Considering the patient’s age, body weight, and dose-related risks such as infection and dyslipidemia, we ultimately opted for a mechanism switch rather than dose escalation.

IL-17 is a key effector cytokine driving neutrophilic inflammation in ACH. Overactivation of the IL-17/Th17 axis directly promotes abnormal keratinocyte proliferation and differentiation and continuously recruits neutrophils to chemotax and aggregate in the epidermis, forming a positive feedback loop of inflammation, leading to the occurrence and persistence of typical lesions such as erythema, scaling, and pustules. Anti-IL-17A monoclonal antibodies specifically bind to IL-17A cytokines in the serum, block their binding to receptors, curtail downstream pro-inflammatory signal amplification, and improve skin lesions (11). Multiple studies have demonstrated that anti-IL-17 monoclonal antibodies are significantly effective in treating ACH (15–17). Based on the pathogenesis of ACH, IL-36 receptor inhibitors also have advantages, as they act more upstream in the pathogenesis, have a rapid onset of action, perform well in controlling acute flares, and have a stronger theoretical basis in patients with IL36RN mutations. The U.S. FDA and the European EMA have approved spesolimab for adult and adolescent GPP patients aged 12 years and older with body weight ≥40 kg; favorable real-world efficacy has also been observed (18). A systematic review and single-arm meta-analysis of 329 patients showed that IL-36 inhibitors achieved a GPPGA response rate of 55% within 2 weeks, significantly better than IL-17 inhibitors. However, regarding relapse rates, the 52-week relapse rate for IL-36 inhibitors was higher than that for IL-17 inhibitors (19). Xeligekimab is a recombinant fully human anti-IL-17A monoclonal antibody approved in China; its core mechanism is specific binding to serum IL-17A, blocking IL-17A binding to the IL-17RA receptor, thereby inhibiting the development of inflammatory responses. Its efficacy in plaque psoriasis is relatively well-established. Due to its fully human nature, it may have lower immunogenicity and a favorable safety profile compared to some similar drugs. Based on the above characteristics of Xeligekimab and considering the patient’s financial constraints, we abandoned the use of IL-36 inhibitors and adopted the strategy of systemic Xeligekimab administration. Given the localized, acral-restricted clinical features of ACH, local injection of IL-17 inhibitors can achieve high drug concentrations in the periungual areas of the hands and feet, maximally inhibit pathogenic IL-17 signaling, and simultaneously avoid systemic IL-17 blockade that would tilt the immune balance toward a Th2 direction. Therefore, after the patient developed immune drift, we adjusted the treatment regimen to fractional injections into the local skin of both hands and feet, avoiding vascular plexuses and severely affected areas. The literature reports cases of local injection of IL-17 or IL-23 inhibitors for focal pustular psoriasis (**Table 1**). This treatment approach has several advantages: by injecting the drug directly into the target lesion area (i.e., periungual areas of hands and feet), high local drug concentrations can be achieved, maximizing inhibition of pathogenic IL-17A signaling and achieving effective control of pustules, erythema, and scaling. Moreover, due to fractionated multi-site administration, the drug acts predominantly locally, with minimal perturbation of the systemic immune system, thereby avoiding systemic adverse effects such as immune drift. Our results showed that after local multi-site injections, the patient’s ACH lesions continuously improved: PPPASI decreased significantly from baseline 24 to 1.2 at week 16, DLQI from 24 to 4, and NAPSI from 160 to 60, with no recurrence of eczematous eruptions. These findings echo a recent report by Wang et al. on an ACH patient who, after failure of systemic ixekizumab, successfully switched to local micro-dose guselkumab injections (21). This study suggests that for localized ACH, local micro-dose biologic injections may be more precise than systemic standard dosing, allowing more efficient delivery of antibodies to target lesions while reducing potential side effects.

**Table 1:** Summary of reported cases of localized pustular psoriasis treated with intralesional IL-17/IL-23 inhibitors.

Title	Authors	Publicati-on year	Age/gender	Biologic drug	Treatment regimen and dosage	Disease	Outcome
Case report: PeriungualXeligekimab injection forrefractory Acrodermatitiscontinua of Hallopeau (6)	Cao P et al.	2026	25/ Female	Xeligekimab	2 mg/lesion by local injection every 2 weeks	ACH	Success
Local injection of different biologics for Palmpolantar Pustulosis: Long-term remission after discontinuation (20)	Dai X et al.	2025	53/ Male	Secukinumab (left), Guselkumab (right)	Secukinumab 0.1 mL (1.5 mg) every 4–6 weeks, Guselkumab 0.1 mL (1 mg) every 4–6 weeks	PPP	Success
Local injection of micro-dose guselkumab for acrodermatitis continua of Hallopeau after failure of systemic ikezikumab treatment (21)	Hou Y et al.	2024	31/ Male	Guselkumab	0.1 mL (1 mg) local injection every 2 weeks, total 4 injections	ACH	Success
Local injection of micro-dose guselkumab for palmoplantar pustulosis after partial failure of systemic ixekizumab treatment (22)	Zhou C et al.	2024	25/ Female	Guselkumab	0.1 mL (1 mg) local injection every 4 weeks, total 4 injections	PPP	Success
Local injection of micro-dose anti-interleukin-17A antibody for palmpolantar pustulosis: A real-world study (23)	Xia R et al.	2023	43.5 ± 16.0/3F, 5M(8 patients)	Ixekizumab	0.1 mL (0.8 mg) local injection every 2–8 weeks, average 6.6 injections	PPP	Success

However, this study has limitations. First, the results from a single case cannot be generalized to the broader ACH population, limiting external validity. Second, the long-term efficacy and optimal dosing interval of local injections require further validation. Additionally, injection depth and dose distribution have not been standardized, making replication difficult. Notably, in clinical practice, attention should be paid to injection site reactions (ISR), including swelling, erythema, pruritus, pain, and induration at the injection site. During the 16-week treatment follow-up of this case, the patient did not experience any ISR; however, patients should be informed that ISR is not uncommon in clinical practice. Most ISRs are mild and self-limiting and can be effectively prevented and managed by optimizing injection technique, rotating injection sites, and patient education. Although clear efficacy and safety were observed, establishing causality requires larger case series or randomized controlled trials.

## Conclusion

For patients with ACH who respond poorly to conventional treatments and JAK inhibitors, IL-17 inhibitors represent an effective therapeutic option. This case indicates that local multi-site injections of Xeligekimab on the hands and feet are significantly effective and can avoid the adverse effect of Th17/Th2 immune imbalance.

## Data Availability

The original contributions presented in the study are included in the article/**Supplementary Material**. Further inquiries can be directed to the corresponding author.
