# Supplementary figures and images for: Case Report: Local injection of an IL-17 inhibitor successfully treats Acrodermatitis continua of Hallopeau and avoids immune shift
Source: Front Immunol. 2026 Jun 9;17:1869496. doi: 10.3389/fimmu.2026.1869496 (PMC13286742; doi:10.3389/fimmu.2026.1869496)

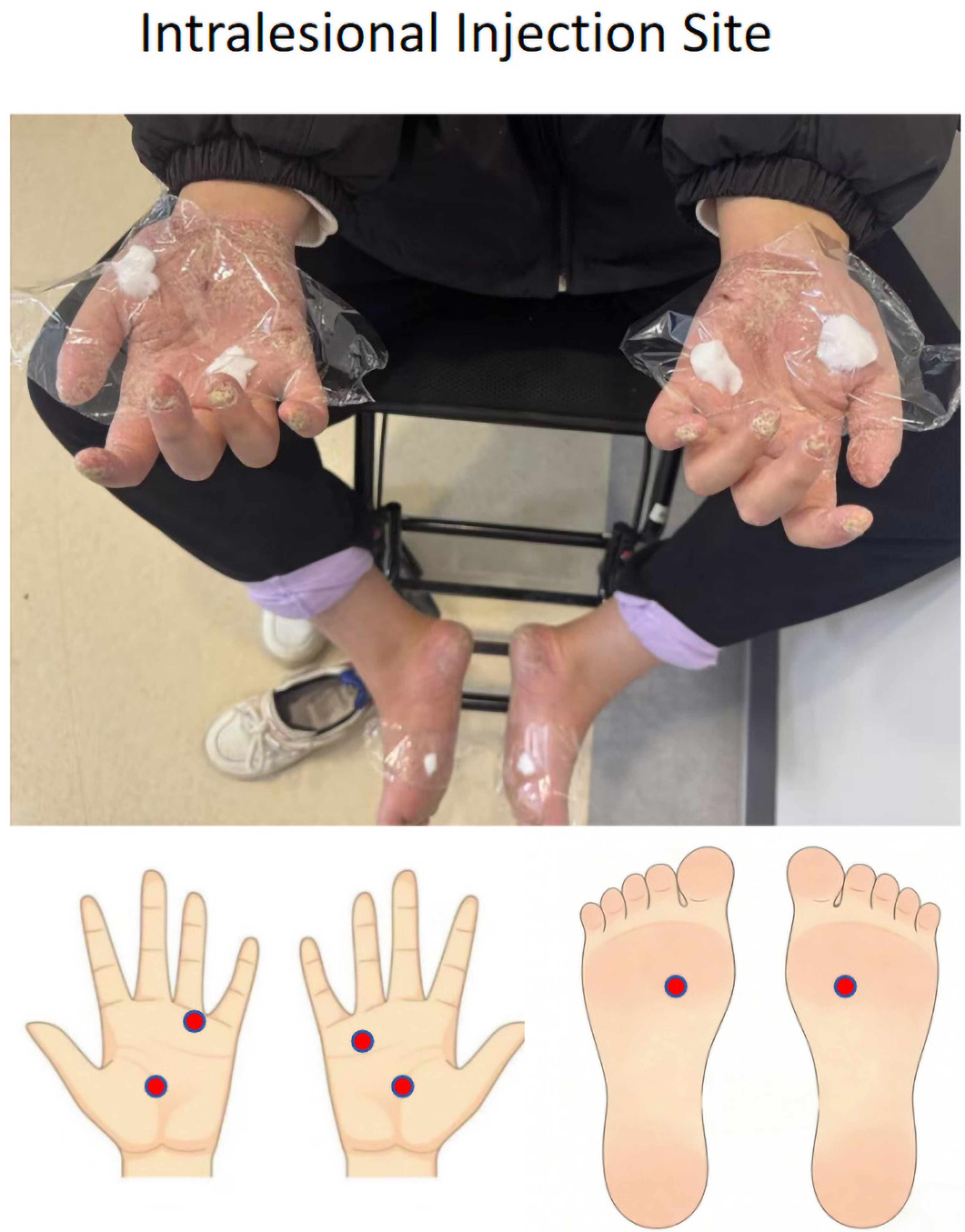

Supplement: Supplementary file 1 [file Image1.jpeg]
